# Supplementary material for: Topological descriptors of the parameter region of multistationarity: Deciding upon connectivity
Source: PLoS Comput Biol. 2023 Mar 24;19(3):e1010970. doi: 10.1371/journal.pcbi.1010970 (PMC10075486; doi:10.1371/journal.pcbi.1010970)
Supplement: Attachment — Submitted filename: Reply.pdf [file pcbi.1010970.s002.pdf]

Reply to the reviewers' comments

We thank the reviewers for the positive comments about the manuscript. We have addressed the reviewers' minor comments, and we detail in what follows our action on each of them.

To ease the consideration of this revision, we have submitted a version of the manuscript with all changes marked in blue.

Action taken on reviewer #1's comments:

- In the abstract and the introduction, it sounds like the number of the connected components for the allosteric reciprocal enzyme regulation network is also computed by the proposed algorithm based on linear programming. In fact, the algorithm only works for the networks that have a connected multistationariy region. So, the authors should clarify this point in the introduction.

  We agree with the reviewer that the formulation was misleading. We have added a few sentences and changed the wording both in the abstract and in the introduction. We have also added a paragraph in the Discussion to emphasize that this example might motivate future work in this direction.

- Maybe the authors can provide more details on how to carry out Step 3 in Algorithm 5 since this is an important step. Now, there is only one sentence "the code is given in the supporting information".

  We see the point that this part was not explained properly. We now explain how to check Step 3 after Theorem 3 (we have also moved the system of inequalities (16)-(18) to after the theorem.

- In the parametrization between the lines 965 and 966, the authors should explain what you mean by the notions $\xi_1, \xi_2, \xi_3, \ldots$ Or, maybe you can simply use $x_1, x_3, x_4\ldots$ (they are clear).

  We have now spelled out what ξ is. We think it is a better idea to keep the notation with ξ, as it connects with the notation of the algorithm.

- Since Theorem 4 is the main theorem of this work, you may want to show how important it is by the experimental results. I understand the discussion in Page 14. But you can still say more on how Theorem 4/Step 4 helps for the examples showing in Table 1.

  We have added a short paragraph to emphasize how important Theorem 4 is. The paragraph is at the end of section "Model reduction for the simplification of the computations".

Action taken on reviewer #2's comments:

- in the formula between line 327 and 328 and in line 332, shouldn't it be $f(\mathbb{R}_{<0})$?

  Fixed.

- in line 997: maybe use c_α instead of c_*?

  Fixed.

Action taken on reviewer #3's comments:

- It turns out that for most biological networks examined by the authors, the region of multistationarity is connected (for only one network there is more than one connected component). It would be good to understand whether the exceptional network has some hidden motif that results in two connected components. In fact, I would see the point in finding further networks (possibly small toy examples) which show multiple connected components. This would go more in future work.

That's a very good point. We have experimented a bit with removing reactions or species, and simplifying the motif, and then we loose the two connected components. We have added a paragraph in the Discussion, and leave further exploration to future investigation.

- Fig.1 caption line 2: "output IS the concentration"?

  Fixed.

- Fig.3 top right corner: "choices" vs. "choice"

  Fixed.

- line 38: 0th vs. 0-th

  Fixed.

- line 39: "Higher order Betti" vs. "Higher Betti"?

  Fixed.

- lines 101 and 127: "paper" vs. "manuscript"

  We changed the word to "paper".

- line 109: "70s" vs. "70ies"

  Fixed.

- line 183: remove dash from "i_j-th" and "j-th"

  Fixed.

- line 207 (and elsewhere): "negative real half-line" vs. "negative real line"

  We changed it to "negative real half-line" throughout the paper.

- line 232: maybe a word is missing from the sentence "We proceed to..."

  Thanks. We removed the word "how".

- line 233: "consists of" vs. "consists in"

  Fixed.

- line 240: "ith" vs. "i-th"

  Fixed.

- line 244: "all reactions producing them" vs. "reaction producing it"

  We changed the text to "all reactions producing them"

- line 259: why is "Methods" slanted?

  No reason. Fixed.

- formula after line 265: i goes from 1 to ℓ

  Fixed.

- line 277: is the term ”consistent” standard? it is called ”dynamically nontrivial” in ”Banaji: Counting CRNs with NAUTY” and subsequent works (to see the equivalence, apply the Farkas Lemma)

  The term “consistent” goes at least back to Angeli-Sontag (2007), and has been used in different papers related to reaction networks. We have added the reference to Angeli-Sontag now in the paper, to illustrate that it is not a word we come up with. The relation to dynamical non-triviality is not that straightforward. Consistent networks are always dynamically nontrivial, but dynamically nontrivial networks do not need to be consistent. For example: a reaction network with stoichiometric matrix $N = (1 \quad 0)$ is dynamically nontrivial but not consistent.

- line 283: ”coordinates” vs. ”coordinate”

  Fixed.

- line 321: it sounds like the rational function might have polynomial denominator (solution: exchange the two sides of the ”or”, and adjust accordingly in the next sentence)

  We changed the text accordingly.

- line 340: do you require that v is nonzero?

  Thanks. Yes, v should be nonzero. Fixed.

- line 347: v=(2,3)?

  Changed to $v = (2, 3)$.

- line 426: not vs. no

  Fixed.

- line 466: ”Since s=3, the negative of the ...”

  We changed the text accordingly.

- line 470: (1,0,0,1,0,1) vs. (1,0,0,1,0,0)

  We changed (1,0,0,1,0,0) to (1,0,0,1,0,1)

- line 535: ”carried out by” vs. ”carried by”?

  Fixed.

- line 549, 703: weakly vs. weak?

  We changed the word to ”weakly”.

- line 602: ”Finally, we consider” vs. ”We have finally consider”

  We changed the sentence to word to ”Finally, we consider”.

- line 686: Theorem 2 holds, because you prove it. Probably you wanted to phrase this sentence differently.

  Thanks. We rephrased the sentence.

- above line 887: an ”=” is missing?

  Fixed.

- line 960: ”no” vs. ”not”

  Fixed.

- line 962: ”state” vs. ”states”

  Fixed.

- line 998: ”=” vs. ”==”

  Fixed.
